# Measuring competition coefficients in an ant community: Implications for intraspecific adaptation load

**DOI:** 10.1002/ecy.70274

**Published:** 2025-12-08

**Authors:** Jumpei Uematsu, Masato Yamamichi, Kazuki Tsuji

**Affiliations:** ^1^ The United Graduate School of Agricultural Sciences Kagoshima University Kagoshima Japan; ^2^ Center for Frontier Research National Institute of Genetics Mishima Japan; ^3^ Genetics Program, Graduate Institute for Advanced Studies SOKENDAI (The Graduate University for Advanced Studies) Mishima Japan; ^4^ School of the Environment The University of Queensland Brisbane Queensland Australia; ^5^ Department of International Health and Medical Anthropology, Institute of Tropical Medicine Nagasaki University Nagasaki Japan; ^6^ Institute for Multidisciplinary Sciences Yokohama National University Yokohama Japan; ^7^ Department of Ecosystem Studies, Graduate School of Agricultural and Life Sciences The University of Tokyo Yayoi Japan; ^8^ Department of Subtropical Agro‐Environmental Sciences University of the Ryukyus Nishihara Japan

**Keywords:** aggression, colony recognition, competition coefficients, density‐dependence, inclusive fitness, social parasitism, species coexistence, stable isotopes

## Abstract

Understanding the stable coexistence of species despite resource competition has been a central topic in ecology. Ant communities are particularly enigmatic as various species coexist despite resource overlap. Community ecology theory predicts stable species coexistence when intraspecific competition is stronger than interspecific competition, but due to their perennial and underground life, competition coefficients of ants have never been rigorously measured in the field. We tackled this problem by studying *Diacamma* cf. *indicum*, which allows for noninvasive mark–recapture of whole colonies. Several ant species coexisted at the study site where *Diacamma* was most abundant, and baiting experiments and stable isotope analyses suggested overlapping food niches. Consistently, per worker brood production of *Diacamma* colonies was significantly negatively correlated with con‐ and heterospecific worker densities within the foraging area, suggesting exploitative competition among the ants. In terms of net population growth, however, the estimated intraspecific competition coefficient was about five times larger than the interspecific competition coefficient. This is possibly because exploitative competition for food occurs both intra‐ and interspecifically, whereas interference competition occurs mostly among conspecifics. Indeed, for *Diacamma* worker survival, there was a significant (nonlinear) negative correlation only with the density of conspecific colonies within the foraging area. This is consistent with the observation that *Diacamma* rarely fought with other species, although it violently attacked conspecific aliens encountered in their nest vicinity. We interpreted these results in light of the recent theory of intraspecific adaptation load. This theory predicts that density‐dependent adaptation to intraspecific conflict can intensify intraspecific competition and act to suppress *per capita* population growth in dominant species, thereby leading to species coexistence with overlapping resources. Our inclusive fitness model suggests that the intraspecific territorial aggression in *Diacamma* may be a counter‐adaptation to intraspecific conflict, that is, brood abduction between conspecific colonies. This aggression pattern can cause the observed density‐dependent worker mortality. Our population dynamic model indicates that such density‐dependent excess mortality acting on dominant competitors can promote stable coexistence with subordinate competitors. Overall, our results support the intraspecific adaptation load theory that aims at integrating behavioral and community ecology to understand how adaptation interacts with population and community dynamics.

## INTRODUCTION

The stable coexistence of competing species, despite the competitive exclusion principle, has been a central enigma in community ecology (Chase & Leibold, 2003; Chesson, 2000; Gause, 1934; Hardin, 1960; Hutchinson, 1961; Levine et al., 2017; Tilman, 1982). Previous theoretical and empirical studies proposed various factors as a potential explanation for stable coexistence including resource differentiation (Chase & Leibold, 2003; Chesson, 2000; Hutchinson, 1959; Tilman, 1982), environmental fluctuations (Armstrong & McGehee, 1980; Chesson & Warner, 1981; Hutchinson, 1961), and the competition–colonization trade‐off (Levins & Culver, 1971; Miller et al., 2024). To explain stable coexistence without these factors, we recently proposed the concept of “intraspecific adaptation load” by focusing on adaptation to intraspecific conflict (Yamamichi et al., 2020). Intraspecific adaptation load is defined as a reduction of growth rates due to adaptation to intraspecific interactions such as sexual selection, sexual conflict, and intraspecific cooperation. Adaptation to intraspecific conflict can improve relative fitness within a population, but at the same time, it can decrease population growth rate (i.e., absolute fitness). If intraspecific adaptation load is density‐dependent (i.e., species with high densities suffer more intensive load), intraspecific competition is intensified and stable coexistence can be promoted (Yamamichi et al., 2023).

Despite the potential importance of intraspecific adaptation load in various organisms, a comprehensive examination of its role for coexistence has yet to be tested. Here, we combine empirical and theoretical approaches to understand the role of intraspecific adaptation load in an ant community. Competition between ants is a promising model system for exploring intraspecific adaptation load, because colony members are generally cooperative but behave aggressively toward conspecific “aliens,” that is, non‐relatives (Tsuji, 2013; Yamamichi et al., 2020). Some ants engage in intraspecific social parasitism, such as nest usurpation and intraspecific slave making, in which they compete for social resources that can only be available for the same species (Hölldobler & Wilson, 1990; Wilson, 1971). Stable coexistence due to intraspecific adaptation load can be clearly demonstrated with the following three components: (1) ecological resource overlap of competing species, (2) larger intraspecific competition coefficients than interspecific competition coefficients despite the resource overlap, and (3) the larger intraspecific competition coefficients arising from intraspecific social/sexual conflict. To tackle these components, we conducted the following investigations: (1) a stable isotope ratio analysis and baiting experiment to clarify how food niches overlap among ant species, (2) rigorous measurements of competition coefficients in the field by noninvasive mark–recapture of an entire ant colony, and (3) behavioral observations to investigate intraspecific conflict as well as mathematical model analyses to understand long‐term ecological and evolutionary dynamics.

**FIGURE 1:**
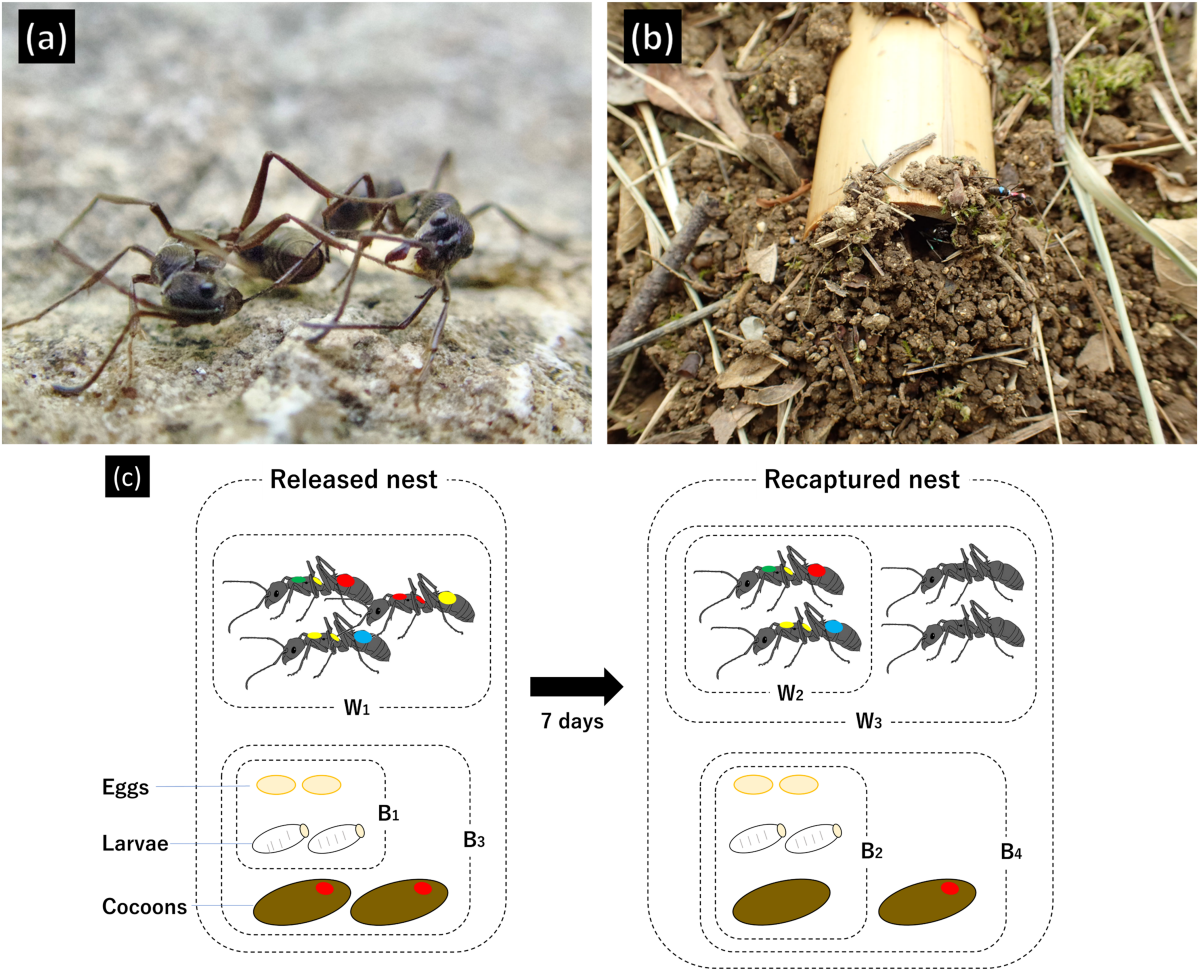
(a) Territorial fights observed between *Diacamma* foragers, (b) a *Diacamma* colony nested in a bamboo tube trap, in which ants cover the nest entrance with grains of soil, and (c) an illustration of how to assess demographic change in a colony by the trapping technique. Photos in panels (a) and (b) were taken by Jumpei Uemastu. Illustrations in panel (c) were drawn by Jumpei Uemastu using MediBang Paint.

The most challenging part is measuring competition coefficients of ants in the wild. Competition coefficients are basic parameters in community ecology that quantify the intensity of competition (Gause, 1934; Hardin, 1960) as the effects of con‐ and heterospecific densities on the instantaneous birth rate, mortality rate, and their sum (i.e., the *per capita* population / biomass growth rate) of the focal species. Stable coexistence is possible when intraspecific competition coefficients are larger than interspecific competition coefficients (Lotka, 1932; Volterra, 1926). Modern coexistence theory uses niche and competitive ability differences, which also rely on competition coefficients (Barabás et al., 2018; Chesson, 2000). However, competition coefficients have never been rigorously quantified in an ant community. In terrestrial ecosystems, these parameters have mostly been estimated in immobile plants (e.g., Adler et al., 2018; Goldberg & Landa, 1991), as it is extremely difficult to estimate the parameters for terrestrial animals in the field. Furthermore, it is difficult to track intra‐colony demography over time in ants, whose nests are perennial and mostly subterranean. Excavation usually kills colonies, and hence a snapshot of demographic data can only be captured once per colony at most. Thus, rigorous empirical data on competition coefficients is largely lacking in ant community ecology. To overcome this, we focused on the Okinawan ant *Diacamma*. This model system allows for the noninvasive mark–recapture of an entire colony by using a colony trapping technique, making it possible to precisely track intracolonial demographic changes in the field (Figure 1).

## MATERIALS AND METHODS

### Study species

The genus *Diacamma* is a large tropical–subtropical ponerine ant, and only *Diacamma* cf. *indicum* is distributed on Okinawa Island, Japan. There are no morphologically distinct castes among adult *Diacamma* females, and all females are monomorphic and wingless. Each colony contains only one inseminated female, called a gamergate, which serves as a functional queen (monogynous). There are 30 to 300 workers in a single colony, which occupies a single nest (i.e., colonies are monodomous) constructed in shallow soil in relatively open areas such as grasslands at forest edges, where workers forage alone (Annagiri, 2021; Tsuji, 2021) for food on the ground within ca. 5 m of the nest (Figure 1; Uematsu et al., 2019). Therefore, a close examination of the impact of ant communities within this spatial range on colony demography would provide a direct estimate of competition coefficients. A point of special interest in the biology of *Diacamma* is the presence of intraspecific slave making via stealing pupae from adjacent colonies (Video S1; Paul & Annagiri, 2019). Perhaps reflecting this, workers violently attack non‐relatives (“aliens”) of the same species that approach their nests (Uematsu et al., 2019; see also *Discussion*). We predict that this can cause intraspecific adaptation load on population growth.

### Study area

Field experiments were conducted from October to December in 2018 and 2019 in an open grassland with pine trees and other shrubs in Sueyoshi Park in Naha, Okinawa (26°13′42.3″ N, 127°42′51.1″ E).

### Colony preparation

Colonies of *D*. cf. *indicum* were collected at the study site by excavating soil (*n* = 5) or by using bamboo ‘traps’ (*n* = 19): When a bamboo tube is buried halfway in the ground, an entire colony of ants will move into its interior (Figure 1b; Fukumoto, 1983). Those colonies were brought back to the laboratory and all adults were individually marked using oil paint markers (Mitsubishi Pencil Co., Ltd., Tokyo, Japan): They were then transferred to a plastic container (44 cm × 30 cm × 7.5 cm high) along with a bamboo tube to serve as the nest (approximately 30 cm long, 5 cm in diameter). All collected colonies were maintained in the laboratory at 25 ± 1°C and 14 L:10 D for 5–22 days, until being released into the field. During captivity, enough mealworms and honey‐water were provided daily to allow the ants to recover from the stress of capture. The number of workers and brood size (the number and total weight of eggs, larvae, and pupae) of the colony were measured on the day before release. Each worker that emerged from a pupa during the laboratory‐rearing period was individually marked. Cocoons were marked with a single dot to distinguish individuals that pupated after release and before recapture.

### Field release and recapture

Each marked colony nested within a bamboo tube was released at a separate point from the collection point in the same area (ca. a hectare) within the same park. The released point was the center of a randomly selected 10‐m square grid, using a map partitioned by a 10‐m mesh. Each grid was used only once. Bamboo tube nests were buried horizontally, with approximately half of the tube below ground, with a small entrance exposed at the ground surface (Figure 1b). Bamboo tube nests were retrieved 1 week later, except in cases where the colony had vacated the tube nest. Immediately after collecting each nest, we waited for 1–2 h to ensure that no foragers were left behind.

After recapture, colony composition was checked in the same way as before release. The individual markings allowed us to assess individual worker survival. Since workers that emerged and cocoons that pupated after the release were unmarked, they could be distinguished from those that were present prior to release.

The survival probability of workers (per worker survival: *S*), brood production per worker (*P*), and net colony growth per worker (*G*) during the 1‐week release period was estimated using the following formulae (see also Figure 1c):

(1) $$S=W_{2}/W_{1},$$

(2) $$P=\left(B_{2}-B_{1}\right)/W_{1},$$

(3) $$G=\left[\left(M\times W_{3}+B_{4}\right)-\left(M\times W_{1}+B_{3}\right)\right]/W_{1},$$

where *W*_1_ is the number of marked workers before release, *W*_2_ is the number of marked workers after recapture (workers that emerged after release were not included), *W*_3_ is the total number of workers after recapture (including workers that emerged after release), *B*_1_ is the total brood weight (the sum of fresh weight of eggs and larvae; we excluded cocoons from *B*_1_ because they are destined to emerge) before release, *B*_2_ is the total brood weight after recapture (the sum of the fresh weights of eggs, larvae, and cocoons; we included newly pupated cocoons here, because they reflect larval growth), *B*_3_ is the total brood weight before release (the sum of eggs, larvae, and cocoons), *B*_4_ is the total brood weight after recapture (the sum of eggs, larvae, and all cocoons), and *M* is the average fresh weight of an individual adult (see Appendix S1: Table S1). The fresh weights of broods were measured with a microbalance (±0.01 mg).

### Estimation of local population density of ants in released plots

The local population densities of conspecific (i.e., *Diacamma*) and heterospecific ants were estimated using the following methods. First, just prior to release, we observed (mapped) the number and location of other *D*. cf. *indicum* colonies within a 5‐m radius (the foraging zone of a *Diacamma* colony (Uematsu et al., 2019)), from the planned colony release point. In addition, nest locations were checked daily during the release period. We defined this as the local conspecific colony density, and only nests present in the 5‐m radius throughout the 7 days (i.e., nest density at recapture) were considered. Note that only 6% of alien colonies vacated the area during the observation period. We did not locate the nests of other ant species, because most entrances of them were highly cryptic and small. In contrast, *Diacamma* builds an obvious crater at the nest entrance (Figure 1c). Immediately after colony recapture, 13 pitfall traps (a 6‐cm diameter, 200‐mL plastic cup containing approximately 100 mL of 10% dishwashing detergent water) were placed in the foraging zone at equal intervals (Appendix S2: Figure S1). The pitfall traps were collected after 24 h, and all ants were identified to the species level. The proxy of biomass of each ant species was estimated as the product of the total number of trapped individuals × the mean wet weight per individual of the species (collected separately; Appendix S1: Table S1). We defined this as the local population density (biomass) of each (con‐ or heterospecific) ant. We also defined the sum of the local population densities (biomass) over the six most abundant non‐*Diacamma* species as the local heterospecific ant density (biomass).

### Statistical analyses of density dependence

The effects of the local densities of con‐ and heterospecific ants on colony performance of *D*. cf. *indicum* in the field were analyzed using a generalized linear mixed model (GLMM) and a linear mixed model (LMM) (R ver. 4.1.0, R Core Team, 2024). There were three objective variables: per worker survival (*S*), brood production per worker (*P*), and net colony growth per worker (*G*). Note that, in the analyses of *S*, the individual worker was treated as the unit; that is, whether an individual was recaptured or not was set as the actual objective variable instead of *S*. Similarly, in the analyses of *P* and *G*, the absolute brood production, *B*_2_ − *B*_1_, and the absolute colony growth, (*M* × *W*_3_ + *B*_4_) − (*M* × *W*_1_ + *B*_3_), were set as the objective variables respectively, and colony size (*W*_1_) was treated as the offset term. The explanatory variables (fixed effects) were colony size (*W*_1_; which was only used in the analysis of worker survival), conspecific density (either the map data for colony density or the pitfall data for worker density), heterospecific density, and their interactions. In addition, colony identity (for worker survival only) and study month (for brood production and net colony growth only) were used as random effects. Since the data (see Results and Discussion) suggested a possible nonlinear effect of the conspecific colony density on worker survival, we included a quadratic term, that is, the square of conspecific colony density as an explanatory variable for worker survival. The other explanatory variables were used as they were, and their interactions with the square of the conspecific colony density were also analyzed. Overdispersion of each variable was tested to choose an appropriate link function. Furthermore, by using the Akaike information criterion (AIC) as an indicator, we selected the most informative combinations of objective variables using the downward (decreasing) stepwise model selection procedure. Heterospecific density was the sum of the biomass of the six non‐*Diacamma* species collected in pitfall traps. In addition, we analyzed species‐by‐species effects (see Appendix S3 for details).

Finally, the parameters of the Lotka–Volterra two‐species competition model (1/*N*_*i*_) (*dN*_*i*_/*dt*) = *r*_*i*_(1 − α_*ii*_ *N*_*i*_ − α_*ij*_ *N*_*j*_) were evaluated by using a linear model. Here, *N*_*i*_ and *N*_*j*_ are population densities of species *i* and *j*, *r*_*i*_ is the intrinsic growth rate of species *i*, α_*ii*_ is the intraspecific competition coefficient of species *i*, and α_*ij*_ is the interspecific competition coefficient that represents the effects of species *j* on the population growth of species *i*. The relative magnitudes of α_*ii*_ and α_*ij*_ are important for the stable coexistence of species *i* and *j*. We evaluated them by using a multiple regression model with the *per worker weight* net colony growth, [(*M* × *W*_3_ + *B*_4_) − (*M* × *W*_1_ + *B*_3_)]/(*M* × *W*_1_), as an objective variable and the con‐ and heterospecific ant densities as explanatory variables. Here, we used the local conspecific colony density (after recapture) for the former density and the local heterospecific ant density (biomass) for the latter one, because these two densities had significant effects on demographic performance of *Diacamma* colonies. On the basis of possible behavioral mechanism, we discuss later why we chose conspecific colony density rather than conspecific worker density to estimate α_*ii*_ (see *Results* and *Discussion*).

## RESULTS

### Community structure

*Diacamma* cf. *indicum* co‐occurred with several other ant species at the study site (Appendix S4: Figure S1). Although the trap data suggest that multiple species were comparable in numerical abundance, *D*. cf. *indicum* was estimated to be dominant in terms of biomass (Appendix S4: Figure S1), because *D*. cf. *indicum* has the largest body size (Appendix S1: Table S1).

### Brood production

**FIGURE 2:**
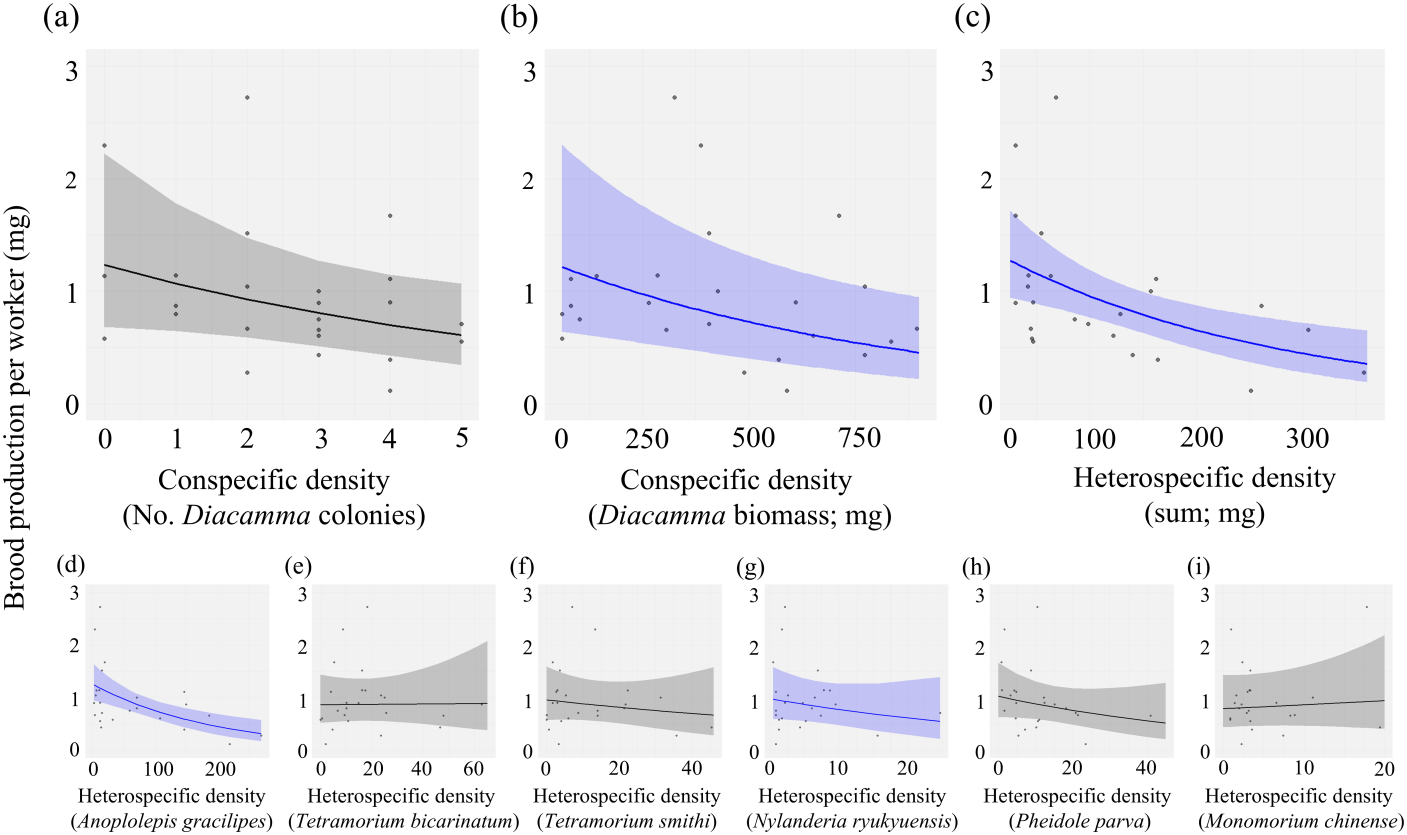
The effect of local population densities of con‐ and heterospecific ants on *Diacamma* brood production. The effects of (a) local conspecific colony density, (b) local conspecific population density (biomass), (c) local heterospecific ant density (biomass), and (d)–(i) local population density (biomass) of each heterospecific ant. Biomass, *x*‐axis in (b)–(i), is expressed in milligrams. Solid lines with colored bands indicate regression lines with 95% confidence intervals. Regression lines for which coefficients were found to be statistically significant are shown in blue. In the analysis, we set the ‘absolute’ brood production, *B*_2_ − *B*_1_, as the objective variable and the colony size, *W*_1_, as the offset term, after all explanatory variables were standardized for technical reasons. To visualize in an easily understandable way, the *y*‐axis shows the brood production per worker, *P* = (*B*_2_ − *B*_1_)/*W*_1_, and the *x*‐axis shows the explanatory variables before standardization. Details of the LMM analysis results can be found in Appendix S3: Table S2.

In the model with the lowest AIC explaining brood production per worker, the local *Diacamma* worker biomass collected in pitfall traps (hereafter, local conspecific density [biomass]), the sum of heterospecific ant biomass collected in pitfall traps (hereafter, local heterospecific ant density [biomass]), their interaction effect, and the random effect (month) were chosen as explanatory variables (see Appendix S3: Table S1 for details). Both local conspecific density (biomass) (coefficient = −0.36, χ^*2*^ = 8.61, df = 1, *p* = 0.0033, Figure 2, Appendix S3: Table S1) and local heterospecific ant density (biomass) (coefficient = −0.29, χ^2^ = 5.85, df = 1, *p* = 0.016; Figure 2c; Appendix S3: Table S1) had significant negative effects on brood production. Furthermore, the interaction effect of con‐ and heterospecific ant biomasses had a significant negative effect on *Diacamma* brood production (coefficient = −0.29, χ^*2*^ = 5.95 5.95, df *= 1, p* = 0.014; Appendix S3: Table S1).

In models in which the effect of heterospecific ant density on *Diacamma* was analyzed on an individual species basis, we found that heterospecific ants' coefficients were negative except for that of *Monomorium chinense* (Figure 2d–i, Appendix S3: Table S1). The coefficients were significant in *Anoplolepis gracilipes* (coefficient = −0.46, χ^*2*^ = 5.57, *df*1, *p* = 0.018; Figure 2d, Appendix S3: Table S2) and in *Nylanderia ryukyuensis* (coefficient = −0.22, χ^*2*^ = 4.81, df1, *p* = 0.028; Figure 2g, Appendix S3: Table S2). The combined effect over the six heterospecific ants was also significant (Fisher's method; χ^*2*^ = 23.4, df12, *p* = 0.024; Appendix S3: Table S1).

We also collected empirical data on food resources. The five dominant ant species (*D*. cf. *indicum*, *Anoplolepis gracilipes*, *Tetramorium bicarinatum*, *Pheidole parva*, and *Monomorium chinense*) active on the surface of the study site were all fed sugar water, mealworms, and tuna in the field (Appendix S5: Table S1). Analysis of stable carbon and nitrogen isotopes suggested considerable diet overlap between species (Appendix S6: Figure S1, Tables S1 and S2). However, nitrogen isotope ratio analysis suggested that the trophic level of *D*. cf. *indicum* is a top predator, although there was a wide range (Appendix S6: Figure S1, Table S1).

### Worker survival

**FIGURE 3:**
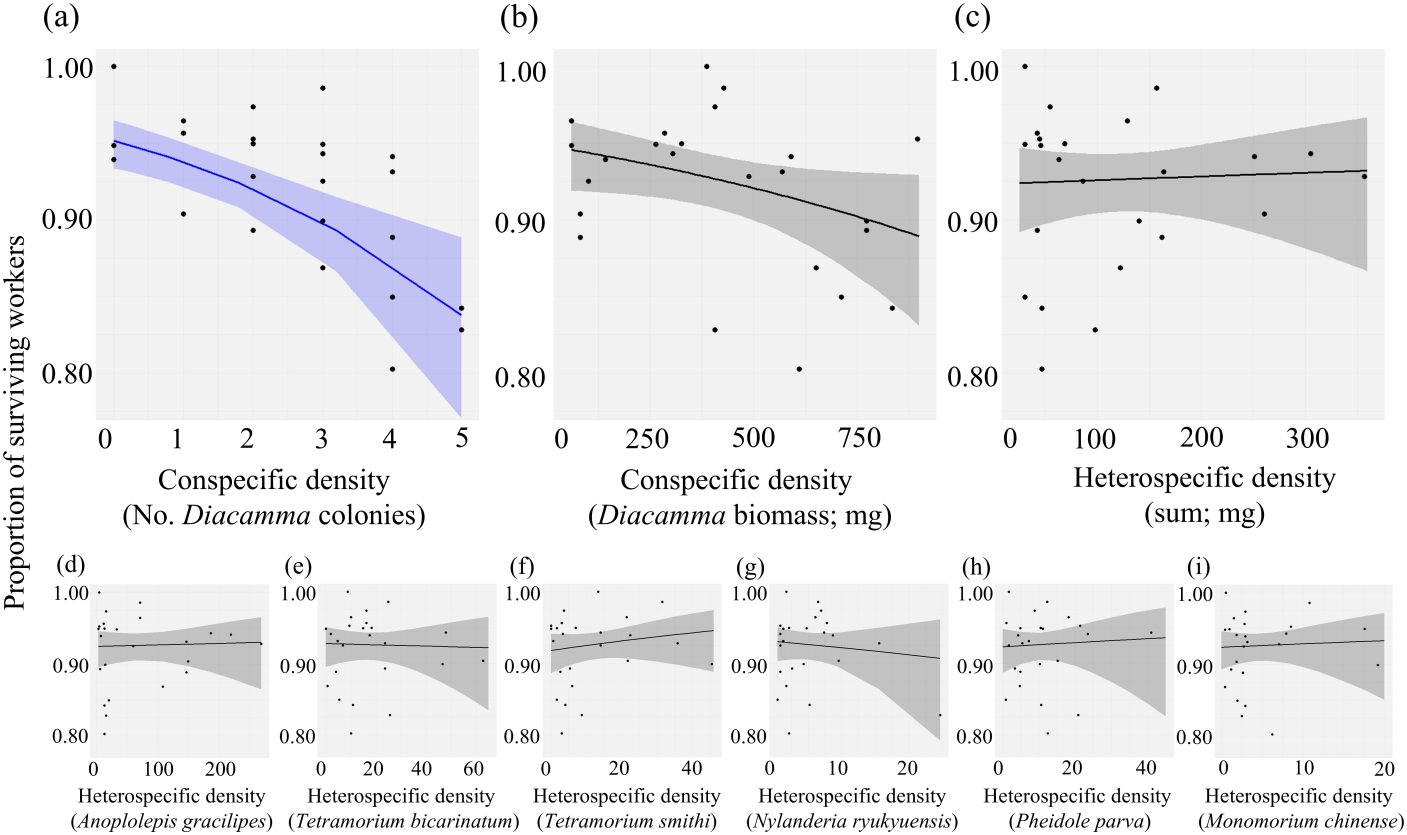
The effect of local population densities of con‐ and heterospecific ants on *Diacamma* worker survival. The effects of (a) local conspecific colony density, (b) local conspecific population density (biomass), (c) local heterospecific ant density (biomass), and (d)–(i) local population density (biomass) of each heterospecific ant. Estimated binomial regression lines with 95% confidence intervals were shown by solid lines with colored bands. Regression lines for which coefficients were found to be statistically significant are shown in blue. In the analysis, an individual was the unit, ‘recaptured or not’ being the objective variable, and all explanatory variables were standardized for technical reasons. To visualize this in an easily understandable way, the *y*‐axis shows the colony‐average survival probability of workers (S) and the *x*‐axis shows the explanatory variable before standardization. Biomass, *x*‐axis in (b)–(i), is expressed in milligrams. Details of the GLMM analysis results can be found in Appendix S3: Table S1.

In the lowest AIC model of worker survival probability in *D*. cf. *indicum*, the following were adopted as explanatory variables: the square of local conspecific colony density, local heterospecific ant density (biomass), their interaction effect, and a random effect (colony ID). The quadratic term of local conspecific colony density had a significant negative effect (coefficient = −0.29, χ^*2*^ = 7.07, df1, *p* = 0.008; Figure 3a, Appendix S3: Table S2). In fact, Figure 3a shows there was an accelerated decline in worker survival as the conspecific colony density increased. The interaction effect of the square of local conspecific colony density and local heterospecific ant density (biomass) was also marginally significant and positive (coefficient = 0.30, χ^*2*^ = 5.10, df1, *p* = 0.024; Appendix S3: Table S2). This implies that the negative effect of the conspecific colony density on worker survival was to some extent relaxed when heterospecific ant density increased in the presence of conspecific colonies. However, the effect of the local heterospecific ant density (biomass) alone was not significant (coefficient = 0.10, χ^*2*^ = 0.80, df1, *p* = 0.37; Appendix S3: Table S2). Random effects (colony ID) were also significant (χ^*2*^ = 4.51, df1, *p* = 0.033; Appendix S3: Table S2). The linear term of the local conspecific colony density and its interaction terms were excluded during the model selection process (Figure 3b). Similarly, during the model selection process, the local conspecific density (biomass) was excluded (Figure 3b) as an explanatory variable in marked contrast to the brood production model. The effect of colony size at release (*W*_1_) was not significant in any of the models and therefore has been excluded. In models in which the effect of heterospecific ant density was analyzed on an individual species basis, no heterospecific ant had a significant effect on *D*. cf. *indicum* worker survival (Figure 3d–i, Appendix S3: Table S2).

The finding that only conspecific density, but not heterospecific densities, showed a pronounced negative effect on worker survival is consistent with the characteristic fighting behavior of *Diacamma* workers. We performed an additional field experiment in which *D*. cf. *indicum* was confronted with alien workers. *D*. cf. *indicum* workers usually showed strong aggression when confronted with a conspecific alien worker (Figure 1a), whereas when confronted with a heterospecific worker, they avoided but rarely attacked the alien (Appendix S7: Table S1). Note that the experiment was conducted near the ant's own nest entrance, where *D*. cf. *indicum* becomes most aggressive. Also remarkably, conspecific alien nest density (map data) but not conspecific alien worker density (pitfall data) showed a significant effect on worker survival, in contrast to the brood production result. This can lead to an important implication for underlying behavioral mechanisms of worker mortality (see *Discussion*).

### Net colony growth and competition coefficients

**FIGURE 4:**
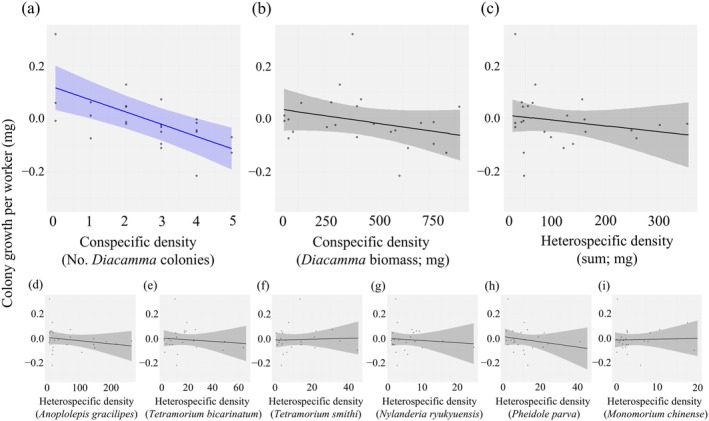
The effect of local population densities of con‐ and heterospecific ants on the net colony growth of *Diacamma*. The effects of (a) local conspecific colony density, (b) local conspecific population density (biomass), (c) local heterospecific ant density (biomass), and (d)–(i) local population density (biomass) of each heterospecific ant. Solid lines with colored bands indicate regression lines with 95% confidence intervals. Regression lines for which coefficients were found to be statistically significant are shown in blue. In the analysis, we set the absolute colony growth, (*M* × *W*_3_ + *B*_4_) − (*M* × *W*_1_ + *B*_3_), as the objective variable and the colony size, *W*_1_, as the offset term, after all explanatory variables were standardized for technical reasons. To visualize in an easily understandable way, the *y*‐axis shows the net colony growth per worker, *G* = [(*M* × *W*_3_ + *B*_4_) − (*M* × *W*_1_ + *B*_3_)]/*W*_1_, and the *x*‐axis shows the explanatory variables before standardization. Biomass, *x*‐axis in (b)–(i), is expressed in milligrams. Details of the LMM analysis results can be found in Appendix S3: Table S3.

In the lowest AIC model that explains the variation in net colony growth of *D*. cf. *indicum*, local conspecific colony density, local heterospecific ant density (biomass), and a random effect (month) were adopted as explanatory variables Appendix S3: Table S3. The local conspecific colony density had a significant negative effect (coefficient = −120, χ^*2*^ = 10.7, df = 1, *p* = 0.0011; Figure 4a, Appendix S3: Table S3). However, no significant effects were detected for other explanatory variables, including total biomass of other species (coefficient = −0.6, χ^*2*^ = 0.32, *df = 1, p* = 0.57; Figure 4b–i, Appendix S3: Table S3). Local conspecific density (biomass) was excluded from the model. Heterospecific ant density on an individual species basis was not significant in any model (Appendix S3: Table S3, Figure 4d–i). The combined effect was not significant (Fisher's method; Appendix S3: Table S3).

Finally, we evaluated the intra‐ and interspecific competition coefficients by using a multiple regression model with the net colony growth *per worker weight*, [(*M* × *W*_3_ + *B*_4_) − (*M* × *W*_1_ + *B*_3_)]/(*M* × *W*_1_), as an objective variable and local conspecific colony density and local density of heterospecific ants (biomass) as explanatory variables. Note that we assumed here a linear effect of density according to the Lotka–Volterra model, although in practice there was some nonlinearity (see *worker survival*). We found that the standardized intra‐ and interspecific coefficients were −0.06 and −0.013, respectively. This means the effect of intraspecific competition was about five times larger than that of interspecific competition. This excessive intraspecific competition coefficient was likely due to territorial aggression by neighboring colonies. When the explanatory variable representing the local conspecific density was changed from colony density (map data) to forager–worker biomass (pitfall trap data, which can more precisely reflect the potential strength of exploitative competition for food), the intra‐ and interspecific competition coefficients became −0.028 and −0.024, respectively. Thus, the ratio shrank from about 4.6 to 1.2.

## DISCUSSION

We achieved precise measurements of intra‐ and interspecific competition coefficients in the ant *Diacamma* cf. *indicum* by focusing on proxies of fitness components (brood production and worker survival) at the colony level. We found evidence of exploitative competition among ants: the tendency for increased densities of both conspecific and heterospecific ants to show negative effects on the brood production of *Diacamma* may reflect exploitative competition for food. It is likely that the more foraging activity of alien ant workers is within the foraging area—regardless of whether they are conspecific or heterospecific—then the less food is available to the focal *Diacamma* colony; this consideration is in line with data showing the ants are all omnivores and that food resources overlap (Appendices [Link], [Link]). The negative effect of conspecific worker density on brood production may also reflect some direct interference, because in *Diacamma* neighboring colonies are known to engage in mutual brood abduction (see below).

We also found evidence of interference competition: worker survival was negatively associated with the conspecific colony density but not with heterospecific ant density (Figure 3, Appendix S3). This seems due to the territorial behavior of *Diacamma*. Although workers forage within a radius of ca. 5 m from the nest, they aggressively defend against conspecific alien incursions only within a radius of 2 m from the nest (Uematsu et al., 2019). In contrast to the aggressive behavior against conspecific aliens, they rarely fight with heterospecific ants encountered but just avoid them (Appendix S7). Furthermore, alien conspecific workers from distant nests are less aggressive and therefore less dangerous to encounter for workers foraging or guarding their own nests (Uematsu et al., 2019). Given the above behavior, high conspecific nest density (rather than high worker density) is more likely to lead to increased incidences of fighting and thus high mortality of foraging workers. Note that this effect is nonlinear (Figure 3a), which can be interpreted as follows. Although foragers search for food widely, they are central place foragers and thus have to pass through the nest entrance on departure and return of foraging trips. Quite naturally and generally ant worker density would be high near the nest entrance. Thus, the proximity of an alien nest would not only increase the probability that an encountered alien is aggressive but would also increase the probability of encountering an alien. These twofold mechanisms might lead to the nonlinear “accelerating” effect.

Incorporating birth and death together, we found that the intraspecific competition coefficient was about five times larger than the interspecific competition coefficient. The predominance of the intraspecific coefficient is possibly because exploitative competition for food occurs both intra‐ and interspecifically, whereas interference competition occurs mostly among conspecifics.

### The role of competition in ant communities

Ants are dominant in most terrestrial ecosystems and make important contributions to biodiversity maintenance and ecosystem functioning (Del Toro et al., 2012; Folgarait, 1998; Hölldobler & Wilson, 1990; Schultheiss et al., 2022; Wilson, 1971). How ants that appear to have similar resource requirements co‐occur has long been discussed (Wilson, 1971, Davidson, 1977a, Davidson, 1977b, Levings & Traniello, 1981, Fellers, 1987, Hölldobler & Wilson, 1990, Ryti & Case, 1992, Adams & Tschinkel, 1995, Morrison, 2000, Andersen, 2008, Boulay et al., 2010, Powell et al., 2011, Parr & Gibb, 2012, Cerdá et al., 2013, Fayle et al., 2015, Adams, 2016, Camarota et al., 2016, Frizzi et al., 2017, De Menezes & Schmidt, 2020, Sheard et al., 2020, McGlinn et al., 2021). Hölldobler and Wilson (1990) stated that “Competition is the hallmark of ant ecology,” which implies that, owing to their ecological dominance and aggressive nature, ubiquitous intra‐ and interspecific interference competition is the key to understanding communities of ants. Many theoretical and empirical studies have been conducted to examine whether ant community structure can truly be explained by competition, using a variety of approaches at different spatial and ecological scales, from intraspecific to metacommunity. For example, previous studies have focused on spatial distribution patterns in relation to environmental gradients (e.g., Davidson, 1977b, a, Levings & Traniello, 1981, Ryti & Case, 1992, Adams & Tschinkel, 1995, Camarota et al., 2016, McGlinn et al., 2021) and on trade‐offs between traits such as resource finding ability, resource holding potentials, niche width, and stress tolerance (e.g., Fellers, 1987; Frizzi et al., 2017; Morrison, 2000; Parr & Gibb, 2012; Sheard et al., 2020). A rare few studies have tracked the survival and growth of nests in the field over years (Ord, 2023; Sundaram et al., 2022; Wiernasz & Cole, 1995) or have employed experimental approaches with nest additions and removals in the field (e.g., Adams & Tschinkel, 2001; Boulay et al., 2010; Fayle et al., 2015). Concerning the role of competition, however, the results are mixed so far (Andersen, 2008, Cerdá et al., 2013, Adams, 2016, see reviews, e.g., Camarota et al., 2016, Sheard et al., 2020, McGlinn et al., 2021).

A rare experimental approach in which excavated colonies were marked and released and then recaptured a year later was described by Boulay et al. (2010), but they only investigated the effect of conspecific density and not of other species' densities. A few other studies have attempted to detect density‐dependent effects on population growth from long‐term tracking of field colonies, because colony growth can be inferred from nest entrance geometry information in some species (Ord, 2023, Sundaram et al., 2022, Wiernasz & Cole, 1995). These data, however, can only provide indirect information for the estimation of competition coefficients. Intra‐colony demography is the product of the birth and death of colony members over time (and migration via colony budding and fusion, if any). It is extremely difficult to quantify these separately in the field. Furthermore, regarding changes in colony density at the population level, there are very few situations in which founding, mortality, and migration of colonies can be strictly distinguished in the field (but for a remarkable exception, see Ingram et al., 2013).

In this study, we partially overcame those difficulties by using the nest trapping technique. The data indicate that, in *Diacamma*, the intraspecific competition outweighs the interspecific competition despite the overlap of food resources with other coexisting species (Appendices [Link], [Link], [Link]). This finding is consistent with the condition of stable species coexistence in community ecology theory (Gause, 1934; Lotka, 1932; Volterra, 1926). This is also in line with the view of Hölldobler and Wilson (1990), that is, competition is the hallmark of ant ecology, as our results suggest that intraspecific interference competition (i.e., aggressive behavior against conspecific alien) is important for species coexistence. In their meta‐analyses in plant communities, Adler et al. (2018) found that intraspecific competition coefficients were four to fivefold higher than interspecific competition coefficients. Interestingly, this ratio is comparable with our current estimation in *Diacamma* ant. Then, an important next question is the underlying mechanism (see the next headline).

Unfortunately, however, we have no information on the competition coefficients of other ant species unlike previous studies on plants, and thus, we must rely on theory. We analyzed ordinary differential equations of the population densities of consumers competing for a single resource (Appendix S8). As is well known (Tilman, 1982), competitive exclusion occurs when different consumers have different minimum resource requirements for population growth (R*) (Appendix S8: Figure S1). However, when we assumed that the dominant species experiences conspecific density‐dependent excess mortality, due to interference competition (just as inferred in *Diacamma*), the theoretical results showed that species coexistence is stable (Appendix S8: Figure S2, Ruan et al., 2007).

Ant colonies are sessile, and hence, competition occurs locally among neighboring colonies. This is similar to plant or sessile animal (e.g., corals, mussels, and barnacles) communities where competition occurs between neighboring individuals, but different from plankton or more mobile animal (e.g., fish, birds, and mammals) communities, in which combinations of interacting individuals are constantly changing due to their mobility (though individual interactions may occur locally). Therefore, in ants, density dependence should be measured at the local scale where a colony interacts with other colonies by focusing on colony‐level performance. This will be of particular importance for understanding the role of spatial coexistence mechanisms in future studies in the context of modern coexistence theory (Barabás et al., 2018; Chesson, 2000) as each colony is discrete and occupies finite amounts of space (Ellner et al., 2024).

### Implication for the intraspecific adaptation load theory

If, as discussed above, the direct cause (proximate mechanism) of the observed density‐dependent worker mortality lies in the territorial behavior of *Diacamma*, an important question is how such behavior has evolved through natural selection (i.e., the ultimate factor). The strong aggression to conspecific aliens encountered in the nest vicinity can be a counter‐adaptation to the brood abduction occurring intraspecifically in *Diacamma* (Video S1; Paul & Annagiri, 2019). In a study of closely related populations in India, stolen broods eventually emerge in nonnatal nests and contribute as workers, so brood abduction is interpreted as a form of intraspecific social parasitism (Paul & Annagiri, 2019). However, in our study site, cannibalism of stolen broods (intra‐guild predation) might have also occurred: the high nitrogen stable isotope ratio in *Diacamma* (Appendix S5: Figure S1, Table S2) may seem due to *Diacamma*'s position in the food chain as a top predator, but this can also be explained by cannibalism within this species. Nest usurpation by a conspecific colony has also been suggested in *Diacamma* (K. Tsuji, unpublished data).

Given such conflict among adjacent nests, we theoretically analyzed the behavior of workers that maximizes inclusive fitness (Appendix S9). The theoretical prediction qualitatively agreed with the observed territorial defense pattern (Uematsu et al., 2019), namely, that aggressiveness changes in a threshold‐like manner depending on the distance from the nest (Appendix S9). The model's output suggests that when the probability of having resources (brood and nest) stolen is high enough—which occurs when encountering an alien near the nest—for a worker it is worth taking the risk of death (also a loss of resources) to fight the alien.

Why are such aggressive interactions observed only within the species? There are at least two possibilities. (1) Broods are social resources as a future work force (rather than as food) that can only be utilized by *Diacamma*. In fact, *Diacamma* markedly differs from other coexisting species in terms of phylogeny and body size, so other species are unlikely to utilize *Diacamma* as “slaves”. The same inference can be applied also to the nests which may have some species‐specific structure. (2) *Diacamma* is the most abundant in the study site, and therefore the most likely rival to the colonies is its own species.

Our results are largely in supportive of the recent theory of intraspecific adaptation load (Yamamichi et al., 2020; Yamamichi et al., 2023). This theory assumes a multilevel‐selection‐like scheme, that is, adaptation to social conflicts can improve relative fitness within a population, but at the same time it can decrease population growth rate (i.e., intraspecific adaptation load). If species with high densities suffer more intensive load, intraspecific competition is intensified and stable coexistence can be promoted (Yamamichi et al., 2023). Our data imply that adaptive territorial behavior may give rise to intraspecific adaptation load to the *Diacamma* population.

It is unclear whether the intraspecific adaptation load theory can be generally applied to explain species coexistence in ants and other social insects. The estimation of the competition coefficients must be less feasible for other ants. However, if one or more of the following patterns discussed in this study are universally found in social insect communities, generalization may be possible in the future. (1) Workers behave more aggressively to an alien when they are near the nest. (2) The exploitation of social resources such as nests and brood primarily occurs within the same species. (3) As a result of (1) and (2), dominant species become more aggressive toward conspecific aliens than to other species. Finally, (4) resources such as food and nest sites overlap between species.

Regarding (1), experiences of naturalists support this idea that social insects most strongly exhibit aggression to aliens encountered near the nest (Tsuji, 2013). This is well documented in wasps (Gamboa et al., 1991; Starks et al., 1998; Venkataraman & Gadagkar, 1992) and has also been reported in ants (Buczkowski & Silverman, 2005; Knaden & Wehner, 2003). Regarding (2), since interspecific social parasitism is known widely in social insects (De La Mora et al., 2020; Rabeling, 2021), we await future empirical research on the relative importance of intra‐ and interspecific exploitation of social resource in general. The prediction (3) contradicts the pattern observed in invasive Argentine ant. In the introduced range, this ant becomes extremely dominant and shows strong hostility toward other ants but not toward own species. However, according to Tsutsui et al. (2000), this is likely a nonadaptive phenomenon resulting from a population genetic bottleneck during invasion into non‐native regions, which leads to the loss of intraspecific genetic variation of the colony identification labels in the introduced population (Tsutsui et al., 2000, Thomas et al., 2006, but see Giraud et al., 2002). Rather, the collapse of species coexistence following the invasion of Argentine ant, which experiences “unnaturally” weak intraspecific competition in the introduced range, may serve as evidence supporting our view that the relative magnitude of intra‐ and interspecific competition is crucial. Regarding (4), we recognize the need for more sophisticated analysis, such as DNA metabarcoding analysis of ant gut contents.

The intraspecific adaptation load theory integrates behavioral ecology and community ecology. There is, however, still a long way to go before it can be rigorously tested. We hope that new ideas and innovative technologies will emerge to surmount these difficulties, just as we have helped to overcome the difficulties of measuring competition coefficients in ants.

## CONFLICT OF INTEREST STATEMENT

We declare we have no competing interests.

## Supporting information


Appendix S1.

Appendix S2.

Appendix S3.

Appendix S4.

Appendix S5.

Appendix S6.

Appendix S7.

Appendix S8.

Appendix S9.

Video S1.

Video S1_Metadata.

## Data Availability

Data and code are available in Dryad at https://doi.org/10.5061/dryad.8pk0p2nwk (Uematsu et al., 2025a) and in Zenodo at https://doi.org/10.5281/zenodo.10824525 (Uematsu et al., 2025b), respectively.
