# Supplementary material for: Measuring competition coefficients in an ant community: Implications for intraspecific adaptation load
Source: Ecology. 2025 Dec 8;106(12):e70274. doi: 10.1002/ecy.70274 (PMC12683613; doi:10.1002/ecy.70274)
Supplement: Supplementary file 1 — Appendix S1. [file ECY-106-e70274-s011.pdf]

Ecology

Appendix S1 for the article: **Measuring competition coefficients in an ant community: Implications for intraspecific adaptation load**

by **Jumpei Uematsu, Masato Yamamichi, and Kazuki Tsuji**

Table S1. Mean per worker wet weight of the seven dominant ant species in the study area. 20 workers per species were collected from two to four colonies. They were weighed to the nearest 0.001 mg and the average wet weight was calculated.

Species	Biomass (mean ± SE mg)
Diacamma* cf. *indicum	**20.34 ± 0.231**
Anoplolepis gracilipes	**1.425 ± 0.0192**
Tetramorium bicarinatum	**0.675 ± 0.0215**
Tetramorium smithi	**0.334 ± 0.00974**
Nylanderia ryukyuensis	**0.433 ± 0.0133**
Pheidole parva	**0.138 ± 0.00599**
Monomorium chinense	**0.109 ± 0.00484**
