# Supplementary material for: Measuring competition coefficients in an ant community: Implications for intraspecific adaptation load
Source: Ecology. 2025 Dec 8;106(12):e70274. doi: 10.1002/ecy.70274 (PMC12683613; doi:10.1002/ecy.70274)
Supplement: Supplementary file 2 — Appendix S2. [file ECY-106-e70274-s008.pdf]

Appendix S2

Ecology

Appendix S2 for the article: **Measuring competition coefficients in an ant community: Implications for intraspecific adaptation load**

by **Jumpei Uematsu, Masato Yamamichi, and Kazuki Tsuji**

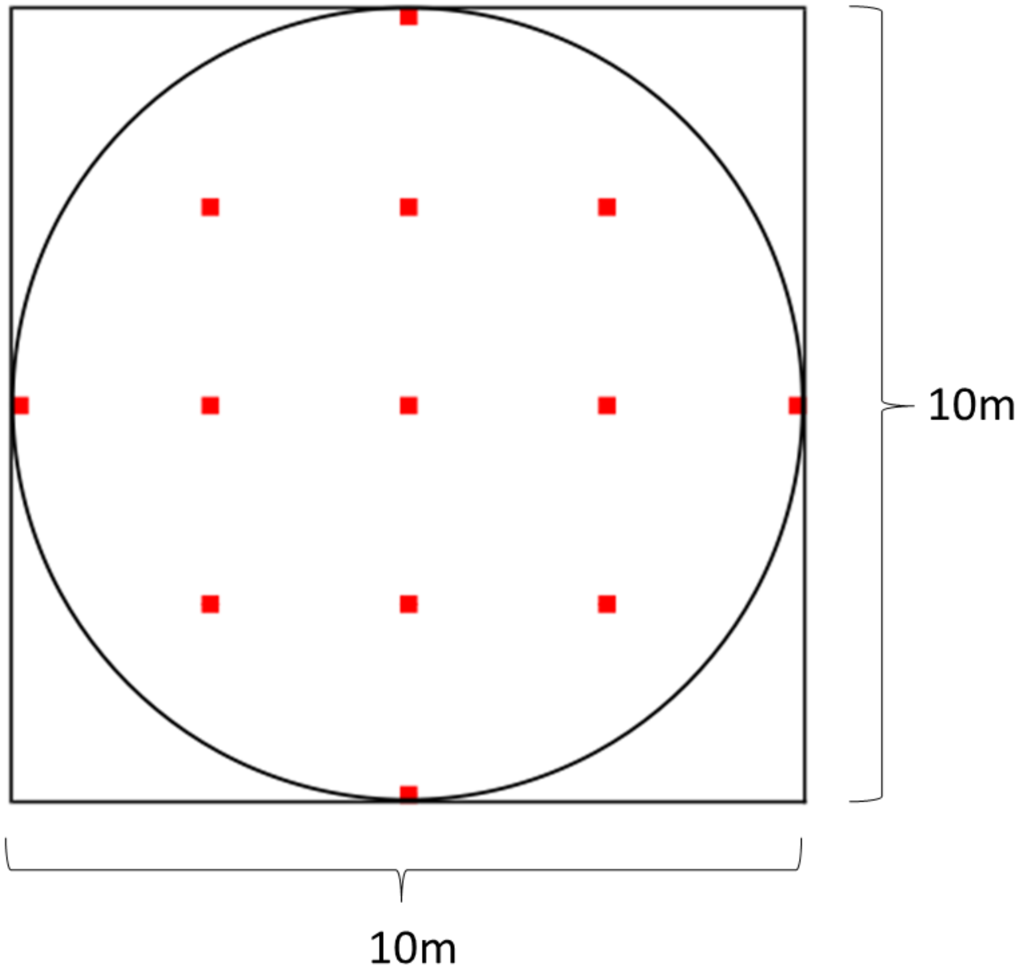

Fig. S1. Schematic presentation of the locations of pitfall traps. Thirteen pitfall traps were placed within a 5-m radius, which is the activity zone of *Diacamma* cf. *indicum* workers [35], from the point of the bamboo tube nest. The trapping was done immediately after recapture of the bamboo tube colony.
