# Supplementary material for: Measuring competition coefficients in an ant community: Implications for intraspecific adaptation load
Source: Ecology. 2025 Dec 8;106(12):e70274. doi: 10.1002/ecy.70274 (PMC12683613; doi:10.1002/ecy.70274)
Supplement: Supplementary file 3 — Appendix S3. [file ECY-106-e70274-s003.pdf]

Ecology

Appendix S3 for the article: **Measuring competition coefficients in an ant community: Implications for intraspecific adaptation load**

by **Jumpei Uematsu, Masato Yamamichi, and Kazuki Tsuji**

Supporting text of statistical analyses for Figs. 2-4 of the main text

All analyses were performed in R (ver. 4.1.0). The local conspecific colony density, the local population density (biomass) of each ant species, the number of surviving workers ($W_1 - W_2$), and the absolute brood production ($B_2 - B_1$) were checked by using the Shapiro–Wilk test for deviation from a normal distribution.

Effect of local population densities on worker survival

Generalized linear mixed models (GLMMs) were used to investigate the effects of conspecific and heterospecific densities on the survival of workers of *Diacamma* cf. *indicum*. Worker survival (i.e., whether an individual was recaptured or not) was set as the objective variable in all models. In the first set of models, the local conspecific colony density, the local heterospecific ant density (biomass), colony size of the focal colony (at the time of release, i.e., W_1; see Methods in the main text), and the interactions of all combinations of the above three variables were initially set as fixed effects. Since the data suggested a possible nonlinear effect of the conspecific colony density on worker survival, we also included a quadratic term, i.e., the square of conspecific colony density as an explanatory variable. The other explanatory variables were used as they were, and their interactions with the square of the conspecific colony density were also analyzed. The values for each fixed effect were standardized and used in the analysis. Furthermore a "colony ID" was set as a random effect that includes putative seasonal variations. A binomial distribution was set for the probability distribution and logit for the link function. The significance of the fixed effects was tested using a chi-square likelihood ratio test.

Assuming the above as the full model, we used the downward stepwise procedure to identify the most informative combinations of explanatory variables that minimize the AIC value.

There were two conspecific ant densities, the local colony density (mapping data) and the estimated biomass (pitfall trap data). Since these values were relatively strongly correlated ($r = 0.437$), we avoided using the two as explanatory variables in the same model. Therefore, in the second set of models we employed the local conspecific density (biomass) as a fixed factor instead of the local conspecific colony density. All other explanatory variables and the calculation procedure were the same as used in the first models. Note that if attacks on alien conspecifics encountered in the vicinity of the nest give rise to an intraspecific adaptation load, worker survival would be correlated with the local conspecific colony density, but not necessarily with the local conspecific density (biomass).

The effect of heterospecific ant density on *D*. cf. *indicum* colony performance may be better analyzed on the basis of each ant species separately. Therefore, a different model was set up for each heterospecific ant. We replaced the local density of heterospecific ants (biomass) with the local density of a single heterospecific ant species in the minimal AIC model in the above analysis; that is, other than this variable, the square of conspecific colony density, their interaction effect, and the random effect (colony ID) were included. There were six different models. The results of the six tests for the effect of heterospecific ant biomass were synthesized using Fisher’s method for combined probability.

The package "lme4" and function “glmer()” in R was used for the GLMM analysis (see File 1 in Uematsu et al. [2025] in Dryad).

Brood production

Generalized linear mixed models (GLMMs) were used to investigate the effect of the densities of conspecific and heterospecific ants on brood production (brood production per worker = Bp; see Methods in the main text) of *D*. cf. *indicum*. The “absolute” brood production ($B_2 - B_1$) was set as the objective variable. As in the above worker survival analysis, we prepared two model

types; one used colony density for the conspecific density and the other used the biomass data instead. In the first set of models, the local conspecific colony density, the local density of heterospecific ants (biomass), and their interaction were set as fixed effects, and the colony size of the colony concerned (W_1) was set as the offset term. Note that the inclusion of the offset term makes the model an analysis of per-worker (W_1) brood production. The fixed effect explanatory variables were standardized and used in the analysis. In addition, month (four categories) was set as a random effect. A gamma distribution was set for the probability distribution and logarithm for the link function. The significance of the fixed effects was tested using a chi-square likelihood ratio test. As in the analysis of worker survival, we also searched for an appropriate model thorough the downward stepwise procedure using AIC as an indicator. We also prepared the second set of models in which the local conspecific density (biomass) was used as a fixed factor instead of the local conspecific colony density. All other explanatory variables and the calculation procedure were the same as in the first models.

Additionally, as in the analysis of worker survival, the effect of heterospecific ants was also analyzed on the basis of each species separately and summed by Fisher's method (for details, see Results in the main text).

The GLMM analysis used the same package and function as for the worker survival analysis above (see File 2 in Uematsu et al. [2025] in Dryad).

Net colony growth

General linear mixed models (LMM) were used to investigate the effect of the densities of conspecific and heterospecific ants on the net colony growth per worker of *D.* cf. *indicum*. The absolute colony growth, $(M \cdot W_3 + B_4) - (M \cdot W_1 + B_3)$, was set as the objective variable. All the other calculation procedures, including the set of explanatory variables and the offset term, were the same as in the analysis of brood production.

Appendix S3

The package "lme4" and function “lmer()” in R was used for the LMM analysis (see File 2 in Uematsu et al. [2025] in Dryad).

Table S1. Results of generalized linear mixed model (GLMM) analysis of brood production. (A) Fixed effects include the local conspecific density (biomass), local heterospecific ant density (biomass), and their interaction. “Month” was set as the random effect. A chi-square likelihood ratio test was used for analysis. (B) The biomass of each heterospecific ant species was used as the explanatory variable, resulting in six models. (C) The statistical significance of the combined effect of the six coefficients for heterospecific ants in (B) was examined using Fisher’s method for combined probability.

**Effect**	**Standard partial regression coefficient**	**χ^2 value**	**df**	***p* value**
**(A) GLMM (brood ~ conspecific density (biomass) + heterospecific density (total biomass) + interaction + random effect)**				
**Conspecific density (biomass)**	**–0.35784**	**8.6097**	**1**	**0.003344**
**Heterospecific density**	**–0.28843**	**5.8589**	**1**	**0.0155**
**Conspecific density (biomass) × heterospecific density**	**–0.29415**	**5.9482**	**1**	**0.01473**
**Month (random effect)**	**—**	**0**	**1**	**1**
**(B) GLMM (brood ~ conspecific density (biomass) + heterospecific density (each species biomass) + interaction + random effect)**				

**Heterospecific density (*Anoplolepis gracilipes*)**	**–0.46054**	**5.5683**	**1**	**0.01829**
**Heterospecific density (*Tetramorium bicarinatum*)**	**–0.09687**	**0.9945**	**1**	**0.3186**
**Heterospecific density (*Tetramorium smithi*)**	**–0.09848**	**0.8262**	**1**	**0.3634**
**Heterospecific density (*Nylanderia ryukyuensis)***	**–0.22076**	**4.8102**	**1**	**0.02829**
**Heterospecific density (*Pheidole parva*)**	**–0.12736**	**2.2362**	**1**	**0.1348**
**Heterospecific density (*Monomorium chinense*)**	**0.04418**	**0.2437**	**1**	**0.6216**
**(C) Fisher's test for combining probabilities over all species-specific analyses of heterospecific biomass**				
**Heterospecific densities (Fisher's test)**	**—**	**23.45336**	**12**	**0.02411453**

Table S2. Results of generalized linear mixed model (GLMM) analysis of worker survival. Fixed effects include square of the local conspecific colony density, local heterospecific ant density (biomass), and their interaction. Colony ID was also included as a random effect. The colony size of released colonies was excluded during the model selection process. A chi-square likelihood ratio test was used for analysis. (A) Total biomass of heterospecific ants collected in pitfall traps was used as the local heterospecific ant density. (B) The biomass of each heterospecific ant species was used, resulting in six models. (C) The statistical significance of the combined effect of the six coefficients for heterospecific ants in (B) was examined using Fisher's method for combined probability.

**Effect**	**Standard partial regression coefficient**	**χ^2 value**	**df**	***p* value**
**(A) GLMM (survival ~ square of conspecific density (colony) + heterospecific density (total biomass) + interaction + random effect)**				
**Square of conspecific density (colony)**	**– 0.28829**	**7.0719**	**1**	**0.00783**
**Heterospecific density**	**0.09555**	**0.7963**	**1**	**0.3722**
**Conspecific density (colony) × heterospecific density**	**0.29972**	**5.0968**	**1**	**0.02397**
**Colony ID (random effect)**	**—**	**4.5129**	**1**	**0.03364**
**(B) GLMM (survival ~ square of conspecific density (colony) + heterospecific density (each species biomass) + interaction + random effect)**				

**Heterospecific density (*Anoplolepis gracilipes*)**	**0.04023**	**0.1595**	**1**	**0.6896**
**Heterospecific density (*Tetramorium bicarinatum*)**	**– 0.06842**	**0.4268**	**1**	**0.5136**
**Heterospecific density (*Tetramorium smithi*)**	**0.02372**	**0.0387**	**1**	**0.8441**
**Heterospecific density (*Nylanderia ryukyuensis*)**	**– 0.05003**	**0.1567**	**1**	**0.6922**
**Heterospecific density (*Pheidole parva*)**	**0.08801**	**0.8297**	**1**	**0.3624**
**Heterospecific density (*Monomorium chinense*)**	**– 0.005197**	**0.0023**	**1**	**0.9616**
**(C) Fisher's test for combining probabilities over all species-specific analyses of heterospecific biomass**				
**Heterospecific densities (Fisher's test)**	**—**	**5.258964**	**12**	**0.9487652**

Table S3. Results of linear mixed model (LMM) analysis of net colony growth. Fixed effects include the local conspecific colony density and the local heterospecific ant density (biomass). A random effect, “month”, is also included. A chi-square likelihood ratio test was used for analysis. (A) Total biomass of heterospecific ants collected in pitfall traps was used as the local heterospecific ant density. (B) The biomass of each heterospecific ant was used, resulting in six models. (C) The statistical significance of the combined effect of the six coefficients for heterospecific ants in (B) was examined using Fisher’s method for combined probability.

**Effect**	**Standard partial regression coefficient**	**χ^2 value**	**df**	***p* value**
**(A) LMM (colony growth ~ conspecific density (colony) + heterospecific density (total biomass) + random effect)**				
**Conspecific density (colony)**	**–123.6712**	**10.671**	**1**	**0.001089**
**Heterospecific density**	**–0.6376**	**0.3202**	**1**	**0.5715**
**Month (random effect)**	**—**	**0**	**1**	**1**
**(B) LMM (colony growth ~ conspecific density (biomass) + heterospecific density (each species biomass) + interaction + random effect)**				
**Heterospecific density (*Anoplolepis gracilipes*)**	**26.30**	**0.2183**	**1**	**0.6403**
**Heterospecific density (*Tetramorium bicarinatum*)**	**–32.70**	**1.1811**	**1**	**0.2771**

**Heterospecific density (*Tetramorium smithi*)**	**–8.61**	**0.0776**	**1**	**0.7805**
**Heterospecific density (*Nylanderia ryukyuensis*)**	**–10.70**	**0.0783**	**1**	**0.7797**
**Heterospecific density (*Pheidole parva*)**	**10.64**	**0.0367**	**1**	**0.8481**
**Heterospecific density (*Monomorium chinense*)**	**30.65**	**1.0183**	**1**	**0.3129**
**(C) Fisher's test for combining probabilities over all species-specific analyses of heterospecific biomass**				
**Heterospecific densities (Fisher's test)**	**—**	**3.560087**	**12**	**0.9901322**

References

Uematsu, J., M. Yamamichi, and K. Tsuji. 2025. "Measuring competition coefficients in an ant community: Implications for intraspecific adaptation load" [Dataset]. Dryad. https://doi.org/10.5061/dryad.8pk0p2nwk
