# Supplementary material for: Measuring competition coefficients in an ant community: Implications for intraspecific adaptation load
Source: Ecology. 2025 Dec 8;106(12):e70274. doi: 10.1002/ecy.70274 (PMC12683613; doi:10.1002/ecy.70274)
Supplement: Supplementary file 4 — Appendix S4. [file ECY-106-e70274-s004.pdf]

Ecology

Appendix S4 for the article: **Measuring competition coefficients in an ant community: Implications for intraspecific adaptation load**

by **Jumpei Uematsu, Masato Yamamichi, and Kazuki Tsuji**

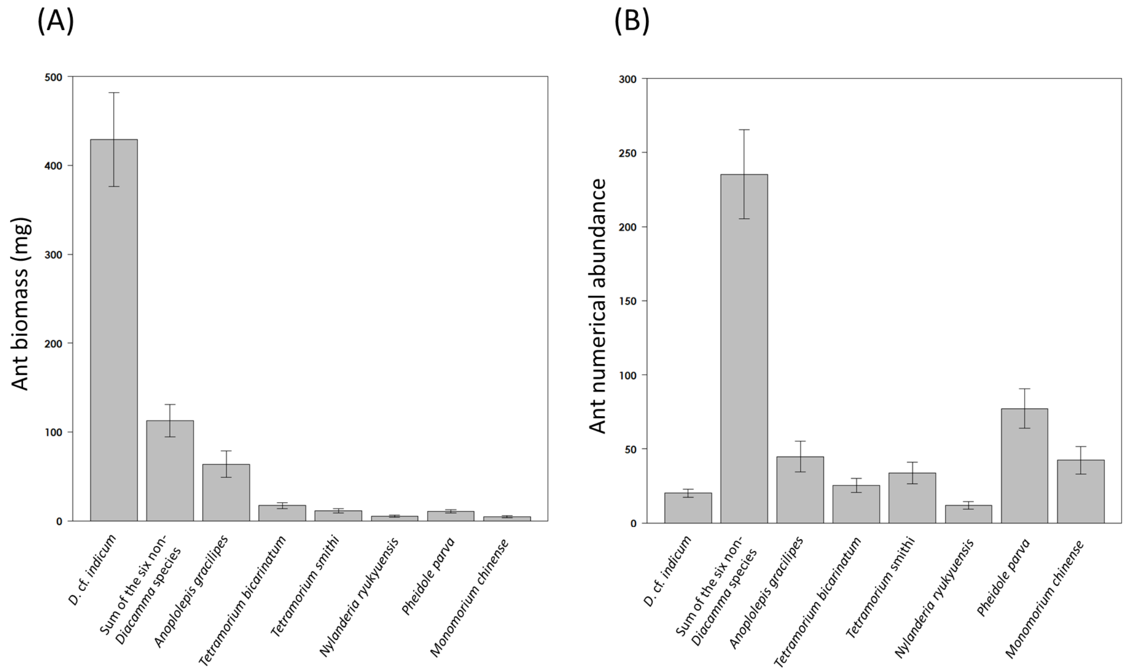

Fig. S1. Comparison of abundance of the major ant species in the study area. (A) Total ant biomass (wet weight) and (B) total number of workers collected in pitfall traps after the mark-and-recapture experiment (see File 6 in Uematsu et al. [2025] in Dryad). Shown are the sum of the values of 13 pitfall traps placed on the foraging area of each *Diacamma* nest (n = 24). Error bars represent standard errors. *Diacamma* cf. *indicum* was the most dominant in the biomass at the study site. See Table S1 of Appendix S1 for biomass per individual worker of each ant species.

References

Uematsu, J., M. Yamamichi, and K. Tsuji. 2025. "Measuring competition coefficients in an ant community: Implications for intraspecific adaptation load" [Dataset]. Dryad. https://doi.org/10.5061/dryad.8pk0p2nwk
