# Supplementary material for: Measuring competition coefficients in an ant community: Implications for intraspecific adaptation load
Source: Ecology. 2025 Dec 8;106(12):e70274. doi: 10.1002/ecy.70274 (PMC12683613; doi:10.1002/ecy.70274)
Supplement: Supplementary file 5 — Appendix S5. [file ECY-106-e70274-s010.pdf]

Ecology

Appendix S5 for the article: **Measuring competition coefficients in an ant community: Implications for intraspecific adaptation load**

by **Jumpei Uematsu, Masato Yamamichi, and Kazuki Tsuji**

Bating experiment

Table S1. The baiting experiment to assess the overlap of the fundamental niches of the five dominant ant species (*Diacamma* cf. *indicum*, *Anoplolepis gracilipes*, *Tetramorium bicarinatum*, *Pheidole parva*, and *Monomorium chinense*) in the study area. The experiment was performed from June 1 to 15, 2020. For each species, baits were placed beside the nest entrance for up to 30 min and workers were observed to see if they foraged the baits (either eating them on the spot or bringing them back to their nest), with “1” indicating the bait was foraged and “0” indicating it was not. The baits used were sugar water, tuna (unsalted), and mealworms. Experiments were repeated with five colonies per species (a–e).

**Species***	**Sugar water**	**Tuna**	**Mealworms**
***Diacamma* cf. *indicum* (a)**	**1**	**1**	**1**
***Diacamma* cf. *indicum* (b)**	**1**	**1**	**1**
***Diacamma* cf. *indicum* (c)**	**1**	**1**	**1**
***Diacamma* cf. *indicum* (d)**	**1**	**1**	**1**
***Diacamma* cf. *indicum* (e)**	**1**	**1**	**1**
***Anoplolepis gracilipes* (a)**	**1**	**1**	**1**
***Anoplolepis gracilipes* (b)**	**1**	**1**	**1**
***Anoplolepis gracilipes* (c)**	**1**	**1**	**1**
***Anoplolepis gracilipes* (d)**	**1**	**1**	**1**

***Anoplolepis gracilipes* (e)**	**1**	**1**	**1**
***Tetramorium bicarinatum* (a)**	**1**	**1**	**1**
***Tetramorium bicarinatum* (b)**	**1**	**1**	**1**
***Tetramorium bicarinatum* (c)**	**1**	**0**	**0**
***Tetramorium bicarinatum* (d)**	**1**	**1**	**1**
***Tetramorium bicarinatum* (e)**	**1**	**1**	**0**
***Pheidole parva* (a)**	**1**	**1**	**1**
***Pheidole parva* (b)**	**1**	**1**	**1**
***Pheidole parva* (c)**	**1**	**1**	**1**
***Pheidole parva* (d)**	**1**	**1**	**1**
***Pheidole parva* (e)**	**1**	**1**	**1**
***Monomorium chinense* (a)**	**1**	**1**	**1**
***Monomorium chinense* (b)**	**1**	**1**	**1**
***Monomorium chinense* (c)**	**1**	**1**	**1**
***Monomorium chinense* (d)**	**1**	**1**	**1**
***Monomorium chinense* (e)**	**1**	**1**	**1**

* In addition to these species, *Tetramorium smithi* and *Nylanderia ryukyuensis* were also collected frequently in the pitfall trap survey and classified as dominant species, but they were excluded from this experiment because it was difficult to identify their nest sites.
