# Supplementary material for: Measuring competition coefficients in an ant community: Implications for intraspecific adaptation load
Source: Ecology. 2025 Dec 8;106(12):e70274. doi: 10.1002/ecy.70274 (PMC12683613; doi:10.1002/ecy.70274)
Supplement: Supplementary file 6 — Appendix S6. [file ECY-106-e70274-s007.pdf]

Ecology

Appendix S6 for the article: **Measuring competition coefficients in an ant community: Implications for intraspecific adaptation load**

by **Jumpei Uematsu, Masato Yamamichi, and Kazuki Tsuji**

STABLE ISOTOPE ANALYSES

Materials and Method

A total of 25 ant species including *D.* cf. *indicum* were collected in this experiment (see file 6 in Uematsu et al. [2025] in Dryad). These 25 species were divided into two groups: those that were almost always present in all 24 quadrats and those that were only rarely collected. We focused on the seven species in the former group: *Anoplolepis gracilipes, Tetramorium bicarinatum, Tetramorium smithi, Nylanderia ryukyuensis, Pheidole parva, Monomorium chinense*, and *D.* cf. *indicum*. We considered these as ants with overlapping spatial niches in terms of foraging areas, that is, potential competitors. A preliminary baiting experiment in June 2020 indicated that these seven ant species accepted and ate all types of foods (honey, tuna, and mealworms) we provisioned (Appendix S5).

The stable isotope ratios of carbon (δ13C) and nitrogen (δ15N) were analyzed to investigate the realized food niche overlap between D. cf. indicum and the other six ant species. Ant samples were collected in August 2021 at the site where the mark-and-recapture experiment was conducted. We collected four colonies of each species with 5–40 individuals per colony to yield a colony sample of 0.3 mg dry weight, the amount required for the analysis. Those samples were dried at 60°C in a drying oven for at least 48 hours and stored in a glass vial. For *T. smithi* and *N. ryukyuensis*, samples collected in pitfall traps with tap water were used for the analysis because it was difficult to locate colonies in the field. The stored samples were transported to Shoko Science Co. for analysis.

Appendix S6

Measurements are reported in delta notation (δ): δ13C and δ15N = [(Rsample/Rstandard) − 1] · 1000, where R is the ratio of the heavy/light isotope content (13C/12C or 15N/14N). Isotope ratios are expressed in per mil (‰) relative to international reference standards VPDB (Vienna PeeDee Belemnite) for carbon and atmospheric nitrogen for nitrogen. To test the differences between *D.* cf. *indicum* and the other ant species, a Brunner-Munzel test was used for δ13C and a Welch test for δ15N.

Results

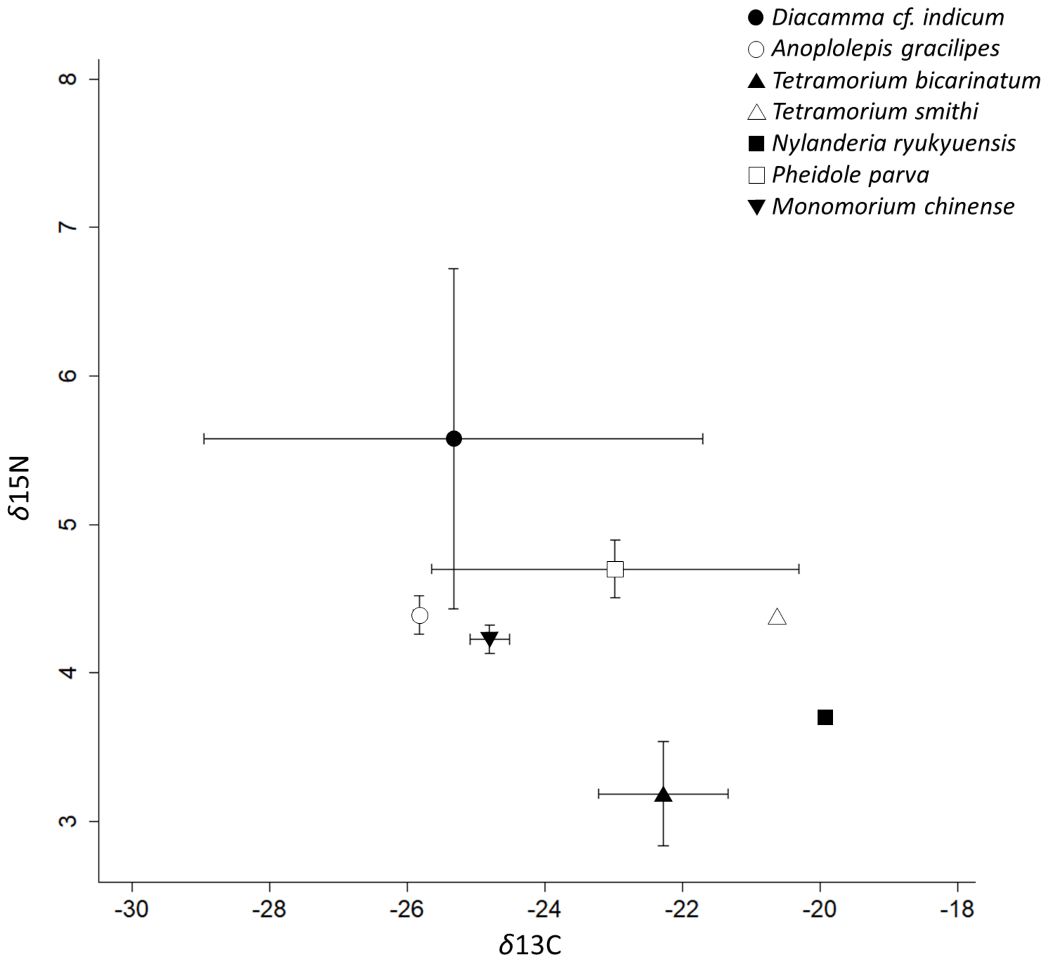

Fig. S1. Biplots of δ^{13}C and δ^{15}N for seven major ant species at the study site. Dots are the species means, and error bars denote 95% confidence intervals. We collected four colonies of each species with 5–40 individuals per colony to yield a colony sample of 0.3 mg dry weight, the amount required for the analysis. For *T. smithi* and *N. ryukyuensis*, samples collected in

pitfall traps. Therefore, these two species are one sample each. Statistical comparison of carbon and nitrogen stable isotope ratios was performed on five species, excluding these two (see Table S1 and Table S2).

Table S1. Statistical comparison of carbon stable isotope ratios ($\delta^{13}C$) of each heterospecific ant species with *Diacamma* cf. *indicum*. The Brunner–Munzel test was used for analysis, and significance levels were adjusted using the Bonferroni method. The *p* values shown were those before the adjustment. The package “lawstat” and the function “brunner.munzel.test()” in R was used to analyze carbon stable isotope (see File 3 in Uematsu et al. [2025] in Dryad).

**Ant species**	***t***	**df**	***p* value**
Anoplolepis gracilipes	**0.37697**	**3.0962**	**0.7306**
Tetramorium bicarinatum	**1.5811**	**3.6585**	**0.1956**
Pheidole parva	**2.5981**	**4.8**	**0.0503**
Monomorium chinense	**1**	**3**	**0.391**

Table S2. Statistical comparison of nitrogen stable isotope ratios ($\delta^{15}N$) of each heterospecific ant species with *Diacamma* cf. *indicum*. The Welch test was used for analysis, and significance levels were adjusted using the Bonferroni method. The package “stats” and function “t.test(x, y, var.equal=F, paired=F)” in R was used for the analysis of nitrogen stable isotope (see File 4 in Uematsu et al. [2025] in Dryad).

**Ant species**	***t***	**df**	***p* value**
Anoplolepis gracilipes	**3.2709**	**3.0776**	**0.04506**
Tetramorium bicarinatum	**6.3398**	**3.5618**	**0.004652***
Pheidole parva	**2.3995**	**3.1666**	**0.09138**
Monomorium chinense	**3.7366**	**3.0411**	**0.03265**

*Significant at the 5% level after Bonferroni correction.

References

Uematsu, J., M. Yamamichi, and K. Tsuji. 2025. "Measuring competition coefficients in an ant community: Implications for intraspecific adaptation load" [Dataset]. Dryad. https://doi.org/10.5061/dryad.8pk0p2nwk
