# Supplementary material for: Measuring competition coefficients in an ant community: Implications for intraspecific adaptation load
Source: Ecology. 2025 Dec 8;106(12):e70274. doi: 10.1002/ecy.70274 (PMC12683613; doi:10.1002/ecy.70274)
Supplement: Supplementary file 7 — Appendix S7. [file ECY-106-e70274-s001.pdf]

Appendix S7

Ecology

Appendix S7 for the article: **Measuring competition coefficients in an ant community: Implications for intraspecific adaptation load**

by **Jumpei Uematsu, Masato Yamamichi, and Kazuki Tsuji**

Responses of *Diacamma* cf. *indicum* workers to conspecific and heterospecific encounters

Methods

To investigate the aggression patterns of *Diacamma* cf. *indicum* workers toward heterospecific ants (*Anoplolepis gracilipes*, *Tetramorium bicarinatum*, *Pheidole parva*, and *Monomorium chinense*) coexisting in the study site, an arena test was performed in which one *D*. cf. *indicum* worker that had just left the nest (focal worker) and one heterospecific worker were placed face to face in a Petri dish placed beside the nest entrance of the focal worker. The experiment was conducted from June 18 to 25, 2020, using five *D*. cf. *indicum* colonies, with each focal worker facing four heterospecific ant species and a conspecific (alien *Diacamma*) colony (control). The heterospecific ants and alien *Diacamma* workers faced by a focal worker were each used only once. We observed each trial for a maximum of 5 min, with an interval of at least 5 min between trials. The strength of aggression was scored as follows: ignore or escape = 0, antennation = 1, bite and/or stab = 2.

Table S1. Responses of *Diacamma* cf. *indicum* workers to conspecific and heterospecific encounters

**Focal worker**	**Test ants**				
***D.* cf. *indicum* colony and individual ID**	***D.* cf. *indicum* (alien conspecific control)**	***Anoplolepis gracilipes***	***Tetramorium bicarinatum***	***Pheidole parva***	***Monomorium chinense***
**Average (SD)**	**1.36 (±0.62)**	**0.28 (±0.45)**	**0.36 (±0.48)**	**0.36 (±0.56)**	**0.40 (±0.57)**
**a-1**	**1**	**1**	**1**	**1**	**0**
**a-2**	**2**	**0**	**0**	**0**	**1**
**a-3**	**1**	**1**	**0**	**0**	**1**
**a-4**	**2**	**0**	**0**	**0**	**0**
**a-5**	**1**	**0**	**0**	**0**	**0**
**b-1**	**2**	**0**	**1**	**0**	**0**
**b-2**	**1**	**0**	**1**	**0**	**0**
**b-3**	**1**	**1**	**0**	**2**	**1**
**b-4**	**2**	**0**	**1**	**0**	**1**
**b-5**	**2**	**0**	**0**	**1**	**0**
**c-1**	**2**	**0**	**0**	**1**	**0**
**c-2**	**1**	**1**	**1**	**1**	**1**
**c-3**	**2**	**0**	**0**	**0**	**0**
**c-4**	**1**	**0**	**0**	**0**	**2**
**c-5**	**1**	**1**	**1**	**0**	**0**

**d-1**	**0**	**0**	**0**	**0**	**1**
**d-2**	**1**	**0**	**1**	**0**	**0**
**d-3**	**1**	**0**	**0**	**1**	**0**
**d-4**	**1**	**0**	**0**	**0**	**0**
**d-5**	**0**	**0**	**0**	**0**	**0**
**e-1**	**2**	**1**	**0**	**0**	**0**
**e-2**	**2**	**0**	**1**	**1**	**0**
**e-3**	**1**	**0**	**0**	**0**	**1**
**e-4**	**2**	**0**	**1**	**0**	**0**
**e-5**	**2**	**1**	**0**	**1**	**1**
