# Supplementary material for: Measuring competition coefficients in an ant community: Implications for intraspecific adaptation load
Source: Ecology. 2025 Dec 8;106(12):e70274. doi: 10.1002/ecy.70274 (PMC12683613; doi:10.1002/ecy.70274)
Supplement: Supplementary file 8 — Appendix S8. [file ECY-106-e70274-s009.pdf]

Ecology

Appendix S8 for the article: **Measuring competition coefficients in an ant community: Implications for intraspecific adaptation load**

by **Jumpei Uematsu, Masato Yamamichi, and Kazuki Tsuji**

Mathematical model of population dynamics

We found that the worker mortality of *Diacamma* cf. *indicum* ants was increased by the number of nearby colonies. We proposed that this is due to aggressive behavior toward conspecifics from different colonies and this "intraspecific adaptation load" (*sensu* Yamamichi et al. 2020) can promote species coexistence.

To explain how the density-dependent mortality rate can affect species coexistence, we consider ordinary differential equations of the consumer population density, N_i (i = 1, 2), competing for a single resource, R:

$$\frac{dN_i}{dt} = N_i\left(c_i a_i R - d_i\right),\ i = 1,2,$$
$$\frac{dR}{dt} = R\left[r\left(1 - \frac{R}{K}\right) - \sum_{i=1}^{2} a_i N_i\right], \tag{S1}$$

where c_i is the conversion efficiency, a_i is the consumption rate, d_i is the consumer mortality (i = 1, 2), and r and K are the intrinsic growth rate and carrying capacity of the resource, respectively. We assume a Holling type I (linear) functional response for the consumers and logistic growth of the resource.

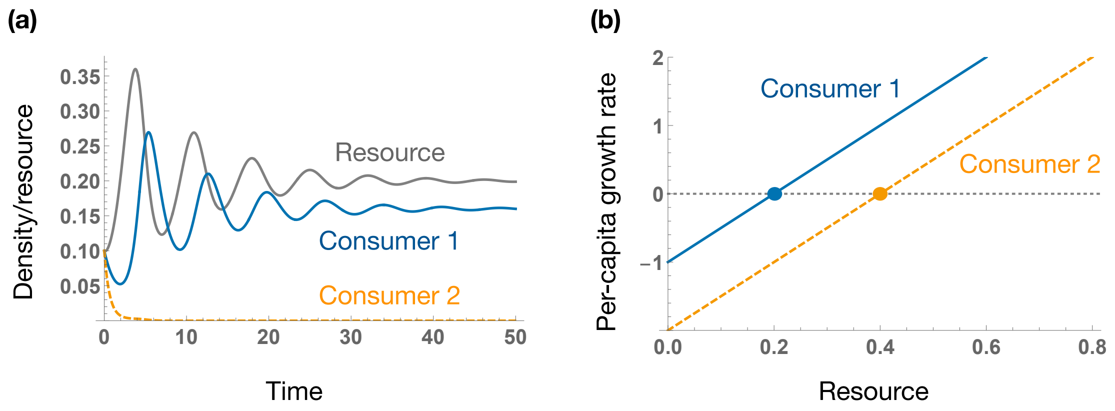

Fig. S1. (a) Dynamics of two consumer populations and resource abundance toward competitive exclusion. (b) Per-capita growth rates of the two consumers. The consumer species with a smaller R^* (= 0.2, indicated by the solid blue lines) excludes the species with a larger R^* (= 0.4, indicated by the dashed orange lines). Parameter values are $a_i = 5$, $d_2 = 2$, and $d_1 = c_i = r = K = 1$ ($i = 1, 2$).

Because there is a single resource (i.e., the single limiting factor: Levin 1970), the two consumer species cannot coexist stably and the species with a smaller R^* will exclude the other species (Fig. S1) (Armstrong and McGehee 1980). Specifically, the minimum resource requirement for population growth, R^*, can be calculated as:

$$R_i^* = \frac{d_i}{c_i a_i},\ i = 1,2. \tag{S2}$$

This is the classical example of the competitive exclusion principle (Gause 1934, Hardin 1960).

Next, we considered the following model with the density-dependent mortality rate of the consumer species 1:

$$\frac{dN_1}{dt} = N_1\left[c_1a_1R - \left(d_{10} + d_{11}N_1\right)\right],$$
$$\frac{dN_2}{dt} = N_2\left(c_2a_2R - d_2\right),$$
$$\frac{dR}{dt} = R\left[r\left(1 - \frac{R}{K}\right) - \sum_{i=1}^{2} a_iN_i\right], \qquad \text{(S3)}$$

where d_{10} is the base mortality rate and d_{11} is the coefficient of the density-dependent mortality rate.

In equilibrium, R of the consumer species 1 can be calculated as:

$$R_1 = \frac{d_{10} + d_{11}N_1}{c_1a_1}. \qquad \text{(S4)}$$

This becomes R^* when $N_1 = 0$ (Eq. S2). When Consumer 2 is absent and Consumer 1 is at its carrying capacity, on the other hand, we can define the resource abundance, $\bar{R}_1$ as:

$$\bar{R}_1 = \frac{K\left(a_1d_{10} + d_{11}\right)}{c_1a_1^2K + d_{11}}, \qquad \text{(S5)}$$

and stable coexistence is possible when $R_1^* < R_2^* < \bar{R}_1$ (Fig. S2). This can be understood from the mutual invasibility (Grainger et al. 2019): when Consumer 1's R^* is smaller than that of Consumer 2, this ensures invasion of Consumer 1 when rare. On the other hand, when $R_2^* < \bar{R}_1$, Consumer 2 can increase when rare, as it has a positive growth rate when Consumer 1 is a resident species and at its carrying capacity. This mutual invasibility ensures stable coexistence of the two consumer species. Mathematica scripts made by Masato Yamamichi were all deposited in Zenodo at https://doi.org/10.5281/zenodo.10824525.

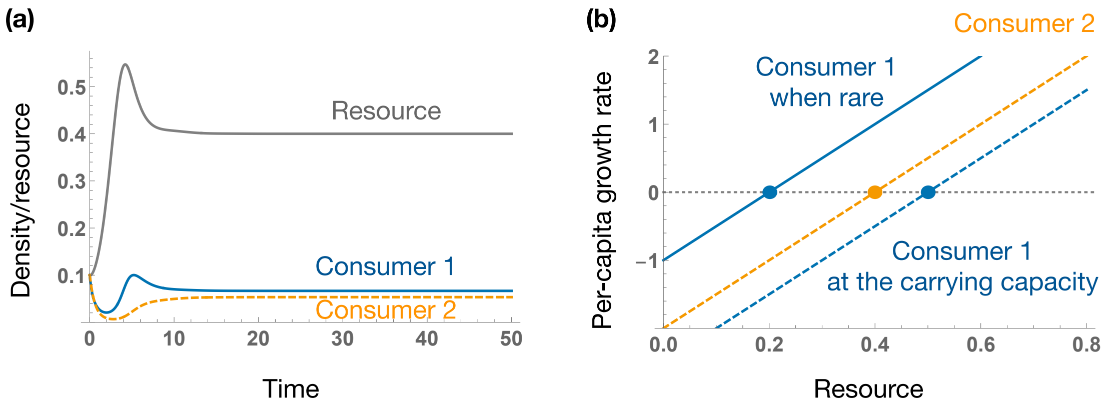

Fig. S2. (a) Dynamics of two consumer populations and resource abundance toward stable coexistence. (b) Per-capita growth rates of the two consumers. Consumer 1, the species with a smaller R^* (indicated by the solid blue lines), decreases its population growth rate (the dashed blue line in b) at the carrying capacity in the absence of Consumer 2. Thus, Consumer 2 (indicated by the dashed orange lines) can increase when rare; that is, Consumer 2's R^* (= 0.4) is smaller than the resource abundance when Consumer 1 is a resident species (= 0.5). Parameter values are $d_{10} = 1$ and $d_{11} = 15$, and other parameter values are the same as in Fig. S1.

References

Armstrong, R. A., and R. McGehee. 1980. Competitive exclusion. The American Naturalist **115**:151-170.

Gause, G. F. 1934. The Struggle for Existence. Williams & Wilkins, Baltimore, MD.

Grainger, T. N., J. M. Levine, and B. Gilbert. 2019. The invasion criterion: A common currency for ecological research. Trends in Ecology & Evolution **34**:925-935.

Hardin, G. 1960. The competitive exclusion principle. science **131**:1292-1297.

Levin, S. A. 1970. Community equilibria and stability, and an extension of the competitive exclusion principle. The American Naturalist **104**:413-423.

Yamamichi, M., D. Kyogoku, R. Iritani, K. Kobayashi, Y. Takahashi, K. Tsurui-Sato, A. Yamawo, S. Dobata, K. Tsuji, and M. Kondoh. 2020. Intraspecific adaptation load: a mechanism for species coexistence. Trends in Ecology & Evolution **35**:897-907.
