# Supplementary material for: Measuring competition coefficients in an ant community: Implications for intraspecific adaptation load
Source: Ecology. 2025 Dec 8;106(12):e70274. doi: 10.1002/ecy.70274 (PMC12683613; doi:10.1002/ecy.70274)
Supplement: Supplementary file 9 — Appendix S9. [file ECY-106-e70274-s006.pdf]

Ecology

Appendix S9 for the article: **Measuring competition coefficients in an ant community: Implications for intraspecific adaptation load**

by **Jumpei Uematsu, Masato Yamamichi, and Kazuki Tsuji**

An inclusive fitness model of territorial behavior

The territorial behavior of *Diacamma* cf. *indicum* from Japan toward alien conspecifics is characterized by strong aggression in the vicinity of the nest and a sudden decrease in aggression at a certain distance (Uematsu et al. 2019). This aggression is likely to defend social resources from kleptoparasitic behavior (brood abduction) by neighboring colonies (see main text and Video S1). Here we present a simple inclusive fitness model explaining the adaptive significance of the observed aggressive territorial behavior.

The Model

Diacamma workers are potentially capable of self-reproduction. However, there is an age-related division of labor in which old workers serve as foragers. The ovarioles of forager-workers are generally inactive; rarely, their fertility is restored when egg-laying opportunities are provided via queen removal (Tsuji 2021; Tsuji et al. 2012). Therefore, it can reasonably be assumed that the survival value of foragers is measured by an indirect component of inclusive fitness through the production of reproductive castes by the colony.

Suppose a *Diacamma* worker engaged in activities outside the nest makes the decision to attack or not to attack upon encountering an alien conspecific. This decision affects the future workforce of the colony to which the focal worker belongs. Here we define workforce as ultimately equal to the expected number of reproductive individuals

that the colony can produce in the future. If the worker attacks, it can fully defend the brood (future workers) from kidnapping, but its colony incurs a cost C in terms of loss of future workforce due to injury or death of the focal worker. If it does not attack, the colony loses future workforce due to brood abduction, of which the expected magnitude $F(d)$ is a function of the distance d between the point of encounter and the nest entrance of the focal worker's colony.

Let r be the expected relatedness of reproductive castes produced by the colony from the worker's point of view. Then, the indirect benefit of aggression equals $r\,F(d)$ and the indirect cost of aggression is $r\,C$. Given that r is always positive, aggression is adaptive when $r\,F(d) > r\,C$ or

$$F(d) > C. \qquad \text{(S1)}$$

Furthermore not to aggress is the favored decision when $r\,F(d) < r\,C$ or

$$F(d) < C. \qquad \text{(S2)}$$

Assuming that $F(d)$ is a monotonically decreasing function of d, the prediction is as shown in Fig. S1a. This prediction agrees qualitatively well with the actual pattern (Fig. S1b).

Fig. S1. (a) The expression pattern of territorial aggression in *Diacamma* cf. *indicum* predicted by the inclusive fitness model. (b) The actual aggression pattern of *D*. cf. *indicum* observed in the field (redrawn using data from Uematsu et al. 2019).

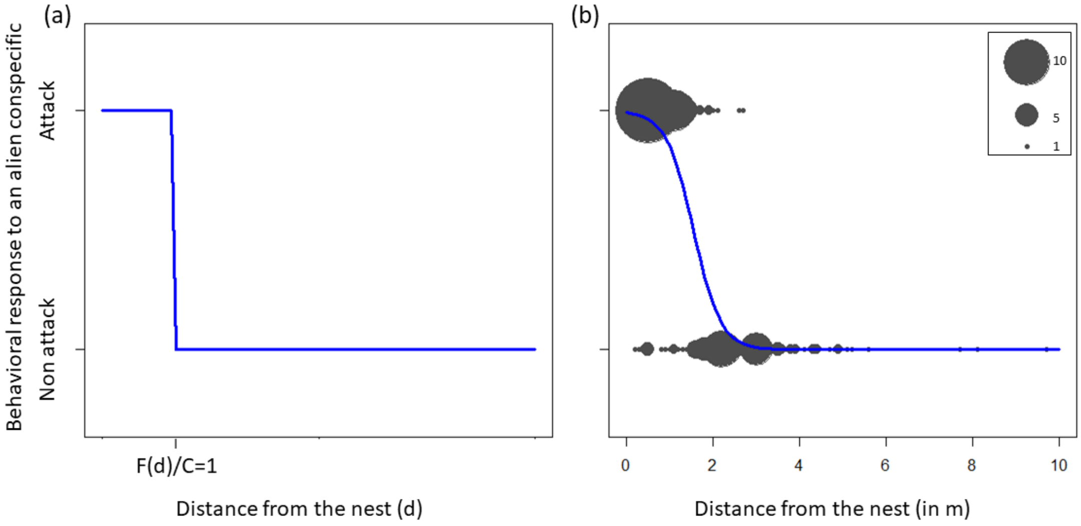

Discussion

The key assumption of the model—that the threat of brood abduction is higher the closer the alien conspecific's location is to the nest—seems reasonable. Everyone would rationally perceive a greater threat when encountering a burglar right in front of one's own home than further away. Although the prediction of our model is in qualitative agreement with the observed phenomenon, quantitative tests are desirable. However, the actual measurement of $F(d)$ and C would be extremely difficult. In the *Diacamma* system, the broods that are kidnapped are primarily pupae (Uematsu et al. 2019; J. Uematsu, pers. obs.), whereas it is the adult foragers themselves that die in the struggle. Both losses may be measurable as biomass reduction, but this would not be a proper assessment of resource loss of the colony, because it should be measured in terms of their contribution to the expected future production of reproductive individuals.

One possible breakthrough is to focus on individual differences among

foragers. The actual pattern (Fig. S1b) has some individual differences, both within and between colonies. One clear prediction of our model is that if there are individual differences in the cost of attack (C), this will affect the aggression pattern. If C is smaller, then we would expect aggressive behavior to occur even when the indirect benefit of the attack is smaller. We assume that the cost of attack is proportional to the loss of the worker's own future labor contribution to the colony due to its own injury or death. If this assumption is correct, we would expect workers with shorter life expectancies to be more aggressive and to attack at greater distances from the nest. This idea should be tested in future studies.

REFERENCES

Tsuji, K. 2021. Reproductive differentiation and conflicts in *Diacamma*: a model system for integrative sociobiology. Asian Myrmecology 13: e013007.

Tsuji, K., N. Kikuta, and T. Kikuchi. 2012. Determination of the cost of worker reproduction via diminished lifespan in the ant Diacamma sp. Evolution 66: 1322–1331.

Uematsu, J., M. Hayashi, H. Shimoji, M. O. Laurent Salazar, and K. Tsuji. 2019. Context dependent aggression toward non-nestmates in the ant *Diacamma* sp. from Japan. J Ethol. 37: 259–264.
