# Supplementary material for: Measuring competition coefficients in an ant community: Implications for intraspecific adaptation load
Source: Ecology. 2025 Dec 8;106(12):e70274. doi: 10.1002/ecy.70274 (PMC12683613; doi:10.1002/ecy.70274)
Supplement: Supplementary file 11 — Video S1_Metadata. [file ECY-106-e70274-s005.pdf]

Video S1 Metadata

Ecology

Measuring competition coefficients in an ant community: Implications for intraspecific adaptation load

Authored by **Jumpei Uematsu, Masato Yamamichi, and Kazuki Tsuji**

Video taken by Jumpei Uematsu

Video S1. A movie of *Diacamma* cf. *indicum* workers stealing pupae from another conspecific colony in the field. Under natural conditions, colonies performing nest relocation are the usual target of brood stealing, but the species also invades neighboring nests to steal pupae. Since it is not feasible to videorecord such scenes, a field experiment was performed to reproduce the situation, following the experimental method of Paul & Annagiri (2019). A *D*. cf. *indicum* colony was housed in a plastic container with plaster of Paris, and all workers were marked in advance. The plastic container was placed on the ground surface of the study site, and the top of the container was removed. Almost immediately after setting the colony, workers from unmarked wild colonies stole the pupae.

Reference

Paul, B., and S. Annagiri. 2019. Caught red-handed: behaviour of brood thieves in an Indian ant. Journal of Experimental Biology **222**:jeb193755.
